# The “Burden” of Childhood Obesity on Bone Health: A Look at Prevention and Treatment

**DOI:** 10.3390/nu17030491

**Published:** 2025-01-29

**Authors:** Ilaria Farella, Mariangela Chiarito, Rossella Vitale, Gabriele D’Amato, Maria Felicia Faienza

**Affiliations:** 1 Department of Medicine and Surgery, LUM University, Casamassima, 70010 Bari, Italy; farella@lum.it; 2 Pediatric Unit, Department of Precision and Regenerative Medicine and Ionian Area, University of Bari “Aldo Moro”, 70124 Bari, Italy; mariangela.chiarito@gmail.com; 3 Giovanni XXIII Pediatric Hospital, University of Bari “Aldo Moro”, 70124 Bari, Italy; r.vitale7@studenti.uniba.it; 4 Neonatal Intensive Care Unit, Di Venere Hospital, 70012 Bari, Italy; gab59it@yahoo.it

**Keywords:** childhood obesity, bone health, adipokines, syndromic obesity, eating disorders, bariatric surgery, GLP-1 receptor agonists

## Abstract

Childhood obesity represents a multifaceted challenge to bone health, influenced by a combination of endocrine, metabolic, and mechanical factors. Excess body fat correlates with an increase in bone mineral density (BMD) yet paradoxically elevates fracture risk due to compromised bone quality and increased mechanical loading on atypical sites. Additionally, subjects with syndromic obesity, as well as individuals with atypical nutritional patterns, including those with eating disorders, show bone fragility through unique genetic and hormonal dysregulations. Emerging evidence underscores the adverse effects of new pharmacological treatments for severe obesity on bone health. Novel drugs, such as glucagon-like peptide-1 (GLP-1) receptor agonists, and bariatric surgery demonstrate potential in achieving weight loss, though limited evidence is available regarding their short- and long-term impacts on skeletal health. This review provides a comprehensive analysis of the mechanisms underlying the impact of childhood obesity on bone health. It critically appraises evidence from in vitro studies, animal models, and clinical research in children with exogenous obesity, syndromic obesity, and eating disorders. It also explores the effects of emerging pharmacological and surgical treatments for severe obesity on skeletal integrity, highlights prevention strategies, and identifies research gaps.

## 1. Introduction

Obesity has become a significant worldwide concern for public health, with the prevalence of pediatric obesity rising dramatically over recent decades [1]. In Europe, nearly one-third of children are classified as overweight or obese, with severe obesity impacting an estimated 800,000 individuals [1]. This trend represents a significant public health concern, as obesity is closely linked to an increased likelihood of developing chronic illnesses (e.g., type 2 diabetes (T2D), cardiovascular diseases) which can manifest already in childhood [2,3]. Despite the established connections between excess body fat and metabolic and cardiovascular complications, a growing concern associated with obesity is its negative impact on bone health, which can have lasting effects on skeletal development [4]. Understanding the impact of obesity-related pathways on bone is critical to addressing obesity’s broader implications beyond its well-known comorbidities. The common culprit of comorbidities linked to obesity is insulin resistance [5]. The restricted ability of adipose tissue to expand results in an overproduction of free fatty acids that build up in the liver and muscle, impairing insulin signaling by triggering the formation of diacylglycerol and ceramides. This process, coupled with dysregulated adipose tissue lipolysis, further exacerbates insulin resistance, leading to the development of conditions such as fatty liver disease, T2D, and metabolic syndrome. Simultaneously, hyperinsulinemia alters mesenchymal stem cell differentiation, favoring adipogenesis at the expense of osteoblastogenesis, thus reducing bone formation [6]. The connection between obesity and bone health is also influenced by mechanical and inflammatory factors. On one hand, increased body weight imposes mechanical loading that can stimulate cortical bone formation. Conversely, the chronic low-grade inflammation linked to obesity undermines this advantage, negatively affecting bone quality, particularly in trabecular bone-rich areas [7]. This inflammation is characterized by high levels of pro-inflammatory cytokines such as TNF-α and IL-6, which activate the receptor activator of nuclear factor kappa-B ligand (RANKL) [8]. RANKL, produced essentially by osteoblastic lineage cells and immune cells during inflammation, promotes osteoclastogenesis by binding to its receptor, RANK, on osteoclast precursors. Osteoprotegerin (OPG), a decoy receptor for RANKL, regulates this pathway and plays a crucial role in protecting bone. Disruption of the RANKL/RANK/OPG axis has been identified as a contributing factor to bone alterations observed in various congenital and acquired pediatric conditions, including obesity [9,10].

This narrative review aims to explore how excess weight may influence bone health in children with exogenous obesity, syndromic obesity, and eating disorders through evidence from in vitro studies, animal models, and clinical research. In particular, we explored (1) the molecular mechanisms underlying bone remodeling and the clinical and experimental aspects of excess weight as determinants of bone health; (2) the effects of emerging treatments, such as GLP-1 receptor agonists and bariatric surgery (BS); (3) the possible prevention strategies.

## 2. Material and Methods

### 2.1. Eligibility Criteria

Manuscripts considered eligible for this review included: i. original published articles and ii. observational or experimental studies.

### 2.2. Information Sources and Search Strategy

The following keywords were searched in PubMed and ClinicalTrials.gov, covering studies published from January 2000 to December 2024: childhood obesity; bone health; adipokines; inflammation; syndromic obesity; anorexia nervosa; autism spectrum disorders; eating disorders; bariatric surgery; GLP-1 receptor agonists

### 2.3. Study Selection

Articles were reviewed with regard to the main topics, i.e., pediatric obesity, bone health, in vitro, in vivo, and human studies.

## 3. In Vitro, In Vivo, and Human Studies

### 3.1. In Vitro Insights: The Role of Pro-Inflammatory Cytokines in Obesity-Induced Bone Loss

**Figure 1:**
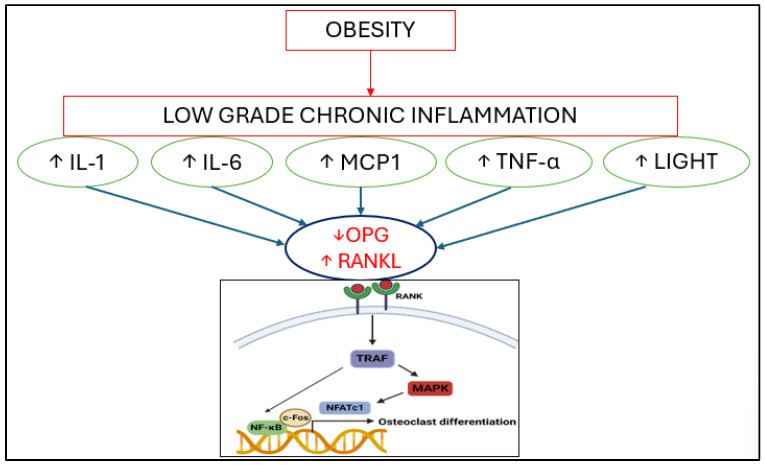
Accumulation of adipose tissue is associated with low-grade chronic inflammation that induces the release of inflammatory factors to activate RANKL-mediated osteoclast differentiation. Abbreviations: IL-1: interleukin 1; IL-6 interleukin 6; MCP1: monocyte chemoattractant protein-1; TNF-α: tumor necrosis factor α; LIGHT: lymphotoxin-like inducible protein that competes with glycoprotein D for herpesvirus entry mediator on T cells; OPG: osteoprotegerin; RANKL: receptor activator of nuclear factor κΒ ligand; TRAF: TNF-receptor-associated factor; NFATc1, nuclear factor of activated T-cells cytoplasmic 1.

Childhood obesity significantly affects bone metabolism through a complex interplay of inflammatory, endocrine, and cellular mechanisms. Adipose-derived cytokines such as TNF-α, IL-6, lymphotoxin-like inducible protein that competes with glycoprotein D for herpesvirus entry mediator on T cells (LIGHT), and monocyte chemoattractant protein-1 (MCP-1) contribute to a state of persistent low-grade inflammation, which influences bone remodeling by promoting osteoclastogenesis and impairing osteoblastogenesis, ultimately favoring bone resorption and compromising bone health, as highlighted by in vitro studies [4,11] (Figure 1).

Adiponectin and leptin have demonstrated distinct and sometimes opposing roles in both in vivo and in vitro studies. Adiponectin is mainly linked to supporting bone formation and improving osteogenesis by encouraging the proliferation and differentiation of osteoblasts while suppressing osteoclastogenesis. On the other hand, leptin influences bone metabolism through intricate and context-specific mechanisms, engaging both central and peripheral pathways.

At the bone marrow level, obesity-induced changes create an inflammatory microenvironment that drives mesenchymal stem cells (MSCs) toward adipogenesis. Da Silva et al. demonstrated this shift, highlighting its connection to an inflammatory bone marrow microenvironment [12]. Likewise, Cortez et al. reported elevated levels of proinflammatory cytokines, including IL-1, IL-6, and TNF-α, in bone marrow MSCs from two-month-old male Wistar rats subjected to a high-fat diet (HFD) [13]. This pro-inflammatory state enhances osteoclast activity while suppressing osteoblast function, contributing to bone loss and disrupted trabecular architecture [14]. TNF-α has the ability to directly stimulate osteoclast formation from bone marrow macrophages, even when RANKL levels are not elevated [15,16]. This cytokine not only synergizes with low levels of RANKL to amplify osteoclastogenesis but can also induce osteoclast differentiation independently under specific conditions, such as the presence of macrophage-colony stimulating factor (M-CSF) [17]. Moreover, TNF-α stimulates stromal cells to produce M-CSF, which enhances the proliferation and differentiation of osteoclast precursors [11]. Interestingly, TNF-α also plays a role in fracture healing, by facilitating mesenchymal stromal cell recruitment and promoting osteogenic differentiation. While low concentrations (1 ng/mL) enhance osteogenesis, higher levels inhibit these processes, demonstrating a biphasic effect. This highlights the complexity of the role of TNF-α, which depends on local inflammatory conditions and concentration levels [18]. Li et al. discovered that IL-6 is essential in driving MSC senescence and contributing to trabecular bone loss in bone marrow stromal cells in wild-type mice fed an HFD [19]. The treatment with IL-6 antibody reduced the expression of senescence markers (p53 and p21) and decreased senescence-associated β-galactosidase activity. Conversely, the addition of recombinant IL-6 to IL-6 knockout mouse bone marrow stromal cells reversed these effects, inducing senescence via the STAT3/p53/p21 signaling pathway. These results suggest that IL-6 amplifies the senescent characteristics of bone marrow stromal cells, disturbs the equilibrium between osteogenesis and adipogenesis, and hastens bone fragility in mice fed an HFD. Kim et al. demonstrated that MCP-1 induces the formation of osteoclast-like cells that are positive for tartrate-resistant acid phosphatase (TRAP), nuclear factor of activated T-cells, and the calcitonin receptor [20]. However, these cells lack bone-resorbing activity in the absence of RANKL. This indicates that MCP-1 is essential in the initial phases of osteoclast differentiation, facilitating cell fusion and the expression of specific markers, while requiring RANKL to finalize the differentiation into functional, bone-resorbing osteoclasts [20]. On the other hand, MCP-1 has been shown to influence bone metabolism by promoting the differentiation of stromal cells into osteoblasts, enhancing osteoblast proliferation, inhibiting osteoclastogenesis, and mediating these effects through both peripheral mechanisms and hypothalamic pathways [21,22]. Adiponectin has been shown in in vitro studies to promote bone formation and osteogenesis in bone marrow stromal cells and MSCs. This is achieved through pathways such as the Adaptor Protein, Phosphotyrosine Interacting with PH Domain and Leucine Zipper 1 Mitogen-Activated Protein Kinase (APPL1-P38 MAPK) and Adiponectin Receptor 1-P38 MAPK, which enhance the production of Bone Morphogenetic Protein 2, a key osteogenic cytokine [23]. Furthermore, adiponectin stimulates osteoblast proliferation and differentiation, increasing alkaline phosphatase activity, type I collagen, and osteocalcin levels, markers of osteoblastic function, through the AdipoR/c-Jun N-terminal Kinase and AdipoR/P38 pathways [24]. Additionally, in vitro studies demonstrate that adiponectin suppresses osteoclastogenesis and bone resorption through APPL1-mediated suppression of protein kinase B [25].

### 3.2. In Vivo Models

Leptin has a multifaceted and debated role in bone metabolism in vivo, with its effects varying significantly depending on the context. Research using leptin-deficient mice, such as Ob/Ob models, highlights its complex role, revealing increased trabecular volume and vertebral bone mass in the spine, coupled with a notable decrease in femoral bone mass and a rise in bone marrow fat within the femur [26,27]. Steppan et al. demonstrated that administering leptin to Ob/Ob mice improved skeletal properties, including increased femur length, systemic bone area, BMD, and bone mineral content (BMC) compared to controls [28]. Subsequent research has corroborated leptin’s capacity to enhance bone mass and quality under specific conditions, with central leptin injections in Ob/Ob mice increasing bone formation, BMD, and muscle mass [26]. Similarly, adiponectin influences bone metabolism in a contradictory manner. Adiponectin knockout mice often show reduced bone mineralization and density compared to wild-type mice [29]. However, some studies suggest that adiponectin deficiency protects against trabecular bone loss induced by ovariectomy and can improve bone quality [30,31]. These contrasting roles of leptin and adiponectin highlight their distinct contributions to bone metabolism and their interplay with pathological states like obesity and estrogen deficiency.

Experimental models of obesity, such as those induced by an HFD, have provided further evidence into the impact of obesity on bone metabolism. While HFD-induced obesity is often associated with increased bone mass due to greater mechanical loading, this does not necessarily correlate with improved bone quality. Histomorphometric analyses reveal reduced bone formation, characterized by decreased osteoblast surface, osteoid surface, and osteoid volume, despite an unchanged mineralization process [32]. Supporting this, Ootsuka et al. demonstrated that TNF-α enhances osteoclastogenesis by activating NF-κB and increasing the expression of RANK, RANKL, and colony-stimulating factor-1 receptor in hyperphagic obese rat models [33]. Feng et al. proposed a model in which IL-6 maintains balanced bone remodeling through the modulation of osteoblast and osteoclast activity. In IL-6-deficient mice, there is increased osteoblast activity, leading to enhanced bone formation and immature trabecular structures. However, despite an increased number of osteoclasts, their resorptive activity is impaired due to reduced expression of osteoclastic markers and higher apoptosis rates. The introduction of an HFD compensates for the lack of IL-6 by reducing osteoblast activity, restoring osteoclast function, and suppressing osteoclast apoptosis. This normalization of bone remodeling counteracts the effects of IL-6 deficiency, leading to a more balanced and mature bone structure. The model highlights IL-6’s dual role and the potential of dietary factors to modulate bone metabolism in its absence [34]. HFD-fed mice exhibit decreased trabecular BMD [35], and while overall bone mass may increase, cortical bone quality and mechanical properties are significantly compromised [36]. Further complicating the picture, ketone body metabolism may exacerbate bone resorption in HFD-induced obesity. Increased expression of acetoacetyl-CoA synthetase, stimulated by IL-6 in osteoclasts, enhances ketone utilization, fueling inflammatory and osteoclastic activity [37]. Encouragingly, treatments like combined supplementation with conjugated linoleic acid and calcium have demonstrated positive outcomes in obese mice, mitigating bone loss and increasing the expression of bone formation markers, such as γ-carboxyglutamate-containing protein 2 and collagen α1. This approach also improves metabolic markers and modulates genes related to energy metabolism, insulin, and leptin receptors [38]. Exercise offers another strategy to mitigate obesity-induced bone loss. Studies reveal that moderate-intensity physical activity preserves bone mass, improves BMD, and enhances trabecular microarchitecture. For instance, voluntary wheel running in obese rats counteracts HFD-induced bone deterioration, improving trabecular density and reducing medullary cavity size [39] (Table 1).

While animal models provide valuable insights into the mechanisms of obesity-induced bone alterations, their relevance to human conditions is limited by several factors. Animal models often exhibit differences in bone architecture, metabolism, and inflammatory responses compared to humans, making direct extrapolation challenging. For instance, the mechanical loading and hormonal environments in rodent models differ significantly from those in children. Furthermore, the rapid skeletal growth in animal models may not fully mimic the gradual skeletal maturation observed in human adolescents. These limitations emphasize the importance of integrating findings from animal models with human studies to ensure clinical relevance.

### 3.3. The Burden of Exogenous Childhood Obesity on Bone Health: Human Studies

The effect of excess weight on bone health in children and adolescents remains intricate and subject to debate, with studies reporting both beneficial and detrimental effects. Children with obesity experience higher mechanical loads on their bones, resulting in larger bone size, enhanced bone strength, higher vertebral density, increased BMC relative to height, greater stature, and accelerated bone maturation compared to their peers with normal weight [40,41,42,43]. Despite this, obesity leads to a systemic pro-inflammatory state that worsens bone health [44]. This pro-inflammatory state is compounded by nutritional deficiencies, leading to a reduced overall bone mass relative to body weight, decreased total bone density, and lower BMC. These conditions predispose to low-velocity fractures [45,46]. Indeed, a study involving 913,178 patients aged 2 to 19 years reported an increased rate of extremity fractures among overweight, moderately obese, and extremely obese individuals, highlighting a potential decline in bone quality [47]. As mentioned above, the elevated levels of pro-inflammatory cytokines, such as TNF-α, IL-1, IL-6, and LIGHT, associated with obesity promote osteoclastogenesis and impair bone formation. These cytokines stimulate the RANK/RANKL pathway, enhancing bone resorption and contributing to skeletal fragility. Supporting this, Gil-Cosano et al. discovered a strong inverse relationship between IL-6 levels and total body-less head BMC in children with obesity [48]. Erazmus et al. demonstrated that overweight and obese children had lower levels of soluble RANKL compared to normal-weight peers, leading to a higher OPG/RANKL ratio. They also found that children in the lowest quartile of RANKL levels had higher BMI and uric acid. These results suggests that excess fat mass alters the OPG/RANKL system and potentially contributes to cardiometabolic and skeletal health complications [49]. Brunetti et al. emphasize the role of LIGHT in driving spontaneous osteoclastogenesis in peripheral blood mononuclear cells (PBMCs) derived from individuals with obesity. Unlike controls, PBMCs from obese individuals were able to form osteoclasts without the need for external stimulation by RANKL or M-CSF. The addition of anti-LIGHT antibodies reduced osteoclast formation in a manner proportional to the dose, confirming LIGHT’s direct involvement in this process [50]. Elevated leptin levels in subjects affected with obesity further exacerbate bone fragility by negatively correlating with OPG and increasing radial cortical porosity and tibial trabecular thickness [51]. In addition, obese subjects have low adiponectin levels and consequently they have decreased osteoblast activity and bone formation [52,53]. The pro-inflammatory microenvironment also influenced the expression of 11β-hydroxysteroid dehydrogenase type 1 (11β-HSD1) in bone, an enzyme responsible for converting inactive cortisone into active cortisol [54]. 11β-HSD1 is expressed in both osteoblasts and adipocytes and its increased activity may be another mechanism by which obesity leads to detrimental effects on bone health [55]. As described above, insulin resistance also impacts bone metabolism. Studies showed a complex relationship between insulin resistance and OPG. Ugur-Altan et al. observed that reduced OPG levels were linked to elevated HOMA-IR values, showing an inverse relationship between OPG and both fasting insulin and glucose levels [56]. Conversely, Suliburska et al. found higher OPG levels in obese adolescents, positively correlating with insulin resistance [57]. Excess fat mass also influences skeletal maturation. Obese children often exhibit accelerated skeletal maturity and advanced bone age beyond their chronological age [58]. Research conducted by Oh et al. revealed that the accelerated bone age seen in children with obesity was strongly associated with body weight, BMI, and waist circumference percentiles [59]. While this accelerated bone growth initially results in increased bone mass and density, it may predispose children to structural anomalies and fragility in adulthood [60]. Despite these endocrine and developmental alterations, mechanical loading from excess weight initially promotes increased bone mass and density. Clark et al. reported higher BMC and BMD in obese children compared to their normal-weight peers, suggesting a positive effect of fat mass on bone accrual during early life [61]. However, this advantage is counterbalanced by poorer bone quality and a higher incidence of fractures, particularly in the lower extremities, as shown by Kessler et al. and Fornari et al. [47,62] (Table 2).

Diets rich in fats and sugary carbonated beverages, but lacking in green leafy vegetables, are commonly linked to obesity and can result in deficiencies of essential micronutrients like vitamin D and calcium [40]. An HFD can interfere with calcium absorption by forming calcium soaps in the intestine, which enhance calcium excretion instead of promoting its absorption [63]. Vitamin D plays a role in regulating leptin and adiponectin levels as well as the production of inflammatory cytokines [64]. Low serum vitamin D levels are frequently observed in children with obesity [65,66]; this is probably due to the sequestration of the liposoluble vitamin D by the subcutaneous fat [66]. Supplementation of vitamin D in obese and overweight subjects may require higher doses than in normal-weight individuals [67,68]. This paradox underscores the fragility underlying increased bone mass in obesity. Engaging in physical activity is crucial for reducing the adverse impact of excess adiposity on bone health. Sedentary lifestyles reduce bone mass accrual and promote tissue absorption by upregulating peroxisome proliferator-activated receptor gamma (PPARγ) in mesenchymal stem cells and RANKL in bone marrow, leading to osteoclast-mediated resorption [69]. Conversely, weight-bearing PA provides mechanical stimuli that promote osteogenesis and inhibits adipogenesis. Behringer’s meta-analysis of 27 studies confirmed that PA, particularly during pubertal growth, enhances BMC accrual [70]. Additionally, irisin, a myokine released during exercise, has been positively associated with BMD in children [71]. These findings emphasize the importance of physical activity in preventing bone fragility and improving skeletal health in children with obesity [72].

## 4. Bone Health in Syndromic Obesity and Overweight Related to Eating Disorders

### 4.1. Bone Health in Syndromic Obesity

**Figure 2:**
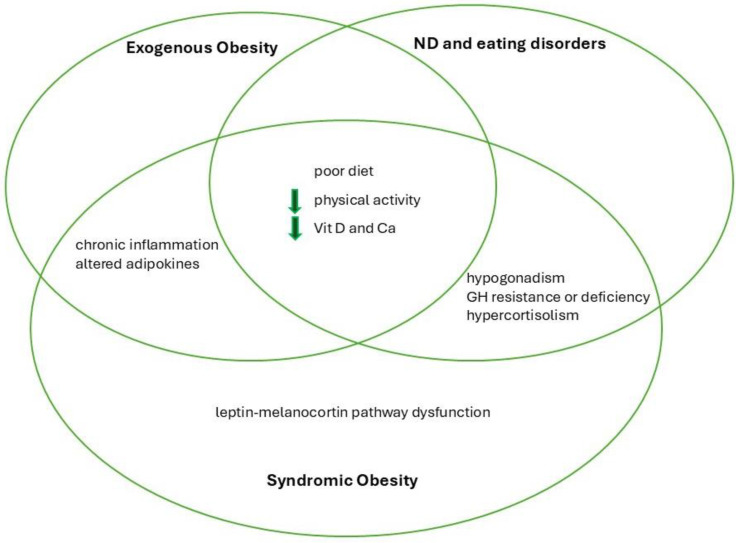
The key pathways by which obesity influences bone health in children and adolescents. The left circle (exogenous obesity) highlights factors such as excessive caloric intake, decreased physical activity, chronic low-grade inflammation (e.g., elevated TNF-α, IL-6, LIGHT), altered adipokine profiles (high leptin/low adiponectin), vitamin D deficiency due to sequestration in adipose tissue or calcium deficiency. The top-right circle (neurodevelopmental disorders/eating disorders) emphasizes the roles of restrictive eating (e.g., autism, anorexia nervosa), endocrine dysfunctions (hypogonadotropic hypogonadism, GH resistance, hypercortisolism), and cognitive or behavioral barriers that limit physical activity. The bottom circle (syndromic/monogenic obesity) illustrates genetic disruptions (e.g., leptin–melanocortin pathway), hypogonadism, growth hormone deficiencies, and poor micronutrient intake. Overlapping regions underscore shared pathways—such as inflammation, endocrine alterations, and nutrient deficiencies—demonstrating how these differing etiologies of obesity can converge to compromise bone health. **Abbreviations**: Ca, calcium; GH, growth hormone; ND, neurodevelopmental; Vit D, vitamin D.

Syndromic obesity is part of a common spectrum of hypothalamic pathologies characterized by severe early onset obesity caused by dysfunction of the leptin–melanocortin pathway that has a pivotal role in satiety/appetite regulation and in energy expenditure [73]. Syndromic obesity is often associated with malformation, dysmorphic features, and often eating disorders, such as impaired satiety and disruptive food-seeking behavior [73]. Bone health in syndromic obesity is still little studied, but it can be impaired by the same mechanisms described in children with exogenous obesity plus other specific mechanisms (Figure 2).

Prader–Willi syndrome (PWS) is the most studied form of syndromic obesity caused by deletion on the paternal chromosome 15q (del15q), maternal uniparental disomy (mUPD), or imprinting defects, with an incidence of about 1/15,000 births. In childhood, individuals with PWS often exhibit normal BMD levels when corrected for their shorter stature [74]. However, during adolescence they may experience a decrease in total BMD and BMC, and in adulthood about 29% to 44% of subjects with PWS may suffer from bone fractures due to the high prevalence of osteoporosis [75]. Possible explanations for the bone impairment include reduced production of sex hormones during puberty and relative growth hormone (GH) deficiency during childhood and adolescence [76]. Additionally, inadequate calcium intake, vitamin D deficiency, limited physical activity, and changes in serum adipokines contribute to disruptions in bone metabolism [77]. Brunetti et al. demonstrated high levels of RANKL and low levels of OPG in both children and adults with PWS compared to sex- and age-matched controls, while sclerostin, an inhibitor of osteoblastogenesis, was found to be higher in PWS children and lower in PWS adults than controls [78]. Consistently with these data, the authors found that BMD was lower in PWS children when compared to healthy controls [78]. Recently, irisin has generated great interest, due to its ability to mimic the effects of physical exercise on bone [71]. Hirsch et al. observed increased levels of salivary irisin in obese individuals with PWS compared to non-obese controls, whereas plasma irisin levels remained similar between the groups [79,80]. Faienza et al. reported that serum irisin levels in individuals with PWS were comparable to those in healthy controls. However, they demonstrate a difference according to genotype underlying the disease [81].

LIGHT levels were found to be significantly higher in both children and adults with PWS than in controls [81]; moreover, serum LIGHT levels negatively correlated with dual-energy X-ray absorptiometry (DXA) parameters.

Alstrom syndrome (ALMS) and Bardet–Biedl syndrome (BBS) are uncommon autosomal recessive disorders, occurring at rates of approximately 1 in 100,000 and 0.7 in 100,000 cases, respectively [82,83]. Both syndromes belong to the category of ciliopathies due to alterations in the molecular structure of the cilium, important for proper functioning of several intracellular signaling pathways [84]. The primary clinical features of both syndromes include early-onset obesity, retinal degeneration progressing to blindness, kidney failure, cardiovascular issues, liver disorders, hypothyroidism, and abnormalities in dental and/or facial development, among others. Patients with BBS frequently exhibit polydactyly, brachydactyly, or syndactyly, as well as intellectual disabilities [82,83]. Bone metabolism can be affected, and osteoporosis is often observed in these subjects even if still little studied [85]. Jeziorni et al. evaluated serum levels of osteocalcin (OC) and urinary deoxypyridinoline (DPD) levels, two bone markers indicating bone formation and bone resorption, respectively, in a cohort of 18 patients (11 with ALMS and 7 with BBS) [86]. They observed reduced levels in the ALMS and BBS groups compared to healthy controls. Additionally, serum OC and urinary DPD values showed a negative correlation with the HOMA-IR index, while a positive correlation was identified between OC and 25-OH vitamin D levels, alongside a negative correlation between RANKL and fasting glucose concentrations [86].

### 4.2. Bone Health in Eating and Neurodevelopmental Disorders

In children and adolescents with eating disorders like anorexia nervosa (AN), bulimia nervosa (BN), pica, or neurodevelopmental conditions like autism and avoidant/restrictive food intake disorder (ARFID), bone accrual is often compromised. While the link between AN or pica and deficiencies in key nutrients such as calcium and vitamin D is well-established, emerging studies highlight the broader impact of these disorders on bone metabolism and structural skeleton integrity during crucial developmental stages. AN, a psychiatric disorder marked by self-induced undernutrition leading to low body weight, profoundly affects bone health, primarily through hormonal disruptions that impair bone metabolism during critical growth phases. This condition triggers significant hormonal adaptations aimed at survival during chronic energy deficiency, albeit at the expense of skeletal integrity. Adolescents with AN, already experiencing dynamic skeletal development, face substantial long-term risks as these hormonal changes result in reduced BMD and microarchitectural damage. A central factor in bone loss in AN is estrogen deficiency caused by hypogonadotropic hypogonadism. While this condition conserves energy by diverting resources from reproduction during nutritional deficits, the resulting hypoestrogenic state is a major driver of bone mass loss. Estrogen deficiency is marked by heightened bone remodeling activity, elevated markers of bone resorption, and a general reduction in bone mass [87,88]. Furthermore, in AN, the duration of amenorrhea correlates with reductions in spinal and femoral neck BMD [87,89]. Acquired GH resistance represents another critical adaptation that reduces energy expenditure in undernutrition states [90] but also significantly contributes to bone mass loss in AN. GH resistance is marked by elevated GH levels that fail to effectively stimulate the production of insulin-like growth factor 1 (IGF-1) [91]. Persistently low IGF-1 levels in AN impair osteoblast function, further hindering bone accrual during adolescence [92]. Additionally, hyperactivation of the hypothalamic–pituitary–adrenal axis leads to elevated cortisol levels, which adversely affect bone health [93]. Cortisol inhibits calcium absorption in the gut, suppresses osteoblast activity, and accelerates osteoclast-mediated bone resorption [94]. Leptin, an anorexigenic hormone secreted by adipose tissue, is also decreased in individuals with AN [95]. Leptin plays a key role in connecting nutrient availability to hypogonadotropic hypogonadism. In women experiencing functional hypothalamic amenorrhea, treatment with recombinant human leptin has been shown to increase LH pulse frequency within two weeks and enhance menstruation recovery rates compared to a placebo [96]. Additionally, leptin therapy raises osteocalcin and bone-specific alkaline phosphatase levels, markers of bone formation [97], and shows a positive association with BMD and bone microarchitecture in AN [98]. However, leptin treatment has notable side effects, including significant weight loss likely due to appetite suppression [99]. The skeletal effects of these hormonal imbalances are evident in the compromised bone microarchitecture of individuals with AN. Approximately 90% of women with AN exhibit BMD values more than 1 SD below the mean for their age [100,101]. Adolescents with AN display reduced cortical thickness, increased porosity, and impaired trabecular connectivity, structural deficits that persist into adulthood and significantly elevate fracture risks [102,103]. Weight recovery remains the cornerstone of treatment and often leads to notable improvements in BMD [104]. However, even with recovery, bone restoration is frequently incomplete, underscoring the irreversible skeletal damage caused during periods of malnutrition [105].

While substantial evidence links AN to impaired bone health through reductions in BMD and BMC, the link between BN and impaired bone health remains less well-documented, with emerging research providing important insights. Studies indicate that BN patients, particularly those with a previous history of AN, exhibit lower BMD, notably at the spine and hip, compared to healthy controls [106]. This indicates that the lasting impact of previous episodes of AN plays a major role in weakening bone health in individuals with BN. Additionally, factors such as low BMI, reduced fat and lean mass, and menstrual irregularities, including amenorrhea, are identified as predictors of reduced BMD in BN. Other studies [107] suggest that purging behaviors and chronic nutritional deficits in BN may further exacerbate risks to bone health. While the connection between BN alone and skeletal compromise remains less clear, the overlap of risk factors with AN underscores the importance of monitoring bone health in BN patients. ARFID also significantly affects growth and bone health during the various stages of development. Research indicates that children and adolescents with ARFID exhibit lower BMD and shorter stature compared to healthy peers, a trend observed across various studies [108,109]. The dietary constraints inherent to ARFID often result in malnutrition, marked by deficiencies in energy and critical nutrients such as calcium, vitamin D, and proteins, which are essential for bone growth and development [110]. Dinkler and colleagues investigated the prevalence and features of ARFID by analyzing data from the Japan Environment and Children’s Study, a large-scale national birth cohort, which included 6633 children aged 4 to 7 years [108]. The study found that children with ARFID were generally shorter than their peers without the disorder. Height differences were also observed among ARFID subtypes, with those classified as A1, A2, or A3—characterized by low body weight, nutritional deficiencies, or reliance on nutritional supplementation—being shorter compared to those in subtype A4, which involves psychosocial impairments without documented nutritional deficiencies. These findings highlight that ARFID’s impact on height varies by subtype, with the greatest deficits seen in subtypes A1–A3. Studies also reveal that the bone deficits in ARFID are comparable to those observed in anorexia nervosa. For instance, research on children and adolescents with ARFID shows that lumbar spine BMD Z-scores are significantly lower and align closely with those of patients diagnosed with AN [109]. Moreover, as in AN, early endocrine disruptions in ARFID, such as altered levels of GH and IGF-1, further compound its effects on bone accrual and overall growth [111]. Pica, characterized by the ongoing consumption of non-food items or raw substances for a minimum duration of one month, has historically been linked to anemia and deficiencies in micronutrients like iron and zinc [112]. While these relationships are well-documented, recent studies explored the association between pica and nutrients critical for bone metabolism, including vitamin D and calcium. Emerging evidence highlights that individuals with pica, particularly adolescents, may be at increased risk of deficiencies in these nutrients, which are essential for proper skeletal development.

A study conducted in northern Sudan involving 344 adolescents demonstrated a significant association between pica symptoms and vitamin D deficiency. Adolescents with pica had median serum vitamin D levels of 17.8 ng/mL, significantly lower than the 21.2 ng/mL observed in their peers without pica (*p* = 0.020). Notably, pica symptoms emerged as an independent predictor of lower vitamin D levels, even after adjusting for confounding factors such as age, sex, and BMI, emphasizing the condition’s impact on vitamin D status [113]. In addition, case series have consistently reported calcium deficiencies in individuals with pica. These deficits are often attributed to the ingestion of non-food substances, such as clay or starch, which bind to calcium in the gastrointestinal tract, reducing its bioavailability. Such deficiencies are particularly concerning in children and adolescents, where calcium is critical for bone mineralization and achieving peak bone mass [114]. Autism spectrum disorder (ASD) is a neurodevelopmental condition that notably affects bone health, especially in children and adolescents. Recent studies indicate that individuals with ASD face challenges in achieving optimal bone development due to various factors, such as nutrient deficiencies, restrictive diets, and reduced levels of physical activity. Dietary selectivity, characterized by a significantly restricted range of food choices and commonly observed in children with ASD [115], contributes to inadequate intake of essential nutrients. Decreased intake of calcium and vitamin D appears to be one of the most consistent findings in children with ASD [116]. This deficiency is further exacerbated by the use of restrictive diets, such as gluten-free and casein-free regimens, which are occasionally suggested for their potential link to symptom reduction [117] but often lack sufficient calcium content. Hediger et al. investigated bone development in 75 boys aged 4–8 years with ASD, focusing on second metacarpal bone cortical thickness. Their findings revealed that although bone cortical thickness increased with age, a notable and progressive divergence from standard reference values began to emerge at the age of six. By age eight, boys with ASD showed an average 25–30% deficit in bone cortical thickness compared to reference medians. Importantly, boys adhering to casein-free diets exhibited nearly double the bone cortical thickness deficits (−18.9%) compared to boys on unrestricted diets (−10.5%). Despite these diets’ potential adverse impact, even boys on unrestricted diets had significantly negative deviations [118]. Neumeyer et al. demonstrated that BMD is significantly lower in peripubertal boys with ASD compared to controls. In an early study utilizing DXA to evaluate 18 boys with ASD and 19 controls aged 8–14 years, researchers observed lower BMD Z-scores at the spine, hip, and femoral neck in boys with ASD. Notably, even after adjusting for maturity and BMI, the differences in BMD at the hip and femoral neck remained, indicating that factors beyond growth and body composition play a role in these deficiencies. The study also highlighted reduced vitamin D intake from both dietary sources and serum levels, alongside lower exercise activity among boys with ASD [119]. In a subsequent study, the same group expanded on these findings by evaluating 25 boys with ASD and 24 typically developing controls, aged 8–17 years. They verified that boys with ASD exhibited BMD Z-scores that were 0.7 to 1.2 standard deviations lower at the lumbar spine, femoral neck, and total hip and in the whole body (excluding the head). Additionally, this research identified key dietary and physical activity differences: boys with ASD consumed 16% fewer calories, with a higher proportion of carbohydrates and significantly less animal protein (37% less) and fat (20% less) than controls. Calcium and phosphorus intake were also lower in the ASD group. Interestingly, just 27% of boys with ASD were categorized as “very physically active”, in contrast to 79% of their typically developing peers [120].

## 5. Prevention at the Core: Strategies for Childhood Obesity and Bone Health

Addressing the complex interplay between childhood obesity and bone health requires a multifaceted approach that encompasses a range of strategies and interventions. This includes early prevention efforts, targeted dietary recommendations, and promotion of physical activity [121]. Growing evidence suggests that the prevention of childhood obesity begins in the preconception period and pregnancy, providing an opportunity to shape the future health outcomes of both mothers and their newborns [122]. Preventive strategies may be further implemented during the breastfeeding period [123]. Human milk’s composition and properties are well-known protective factors [124], and lower-protein formulas are associated with a lower risk of obesity at 6 years [125]. During the postweaning period, diets low in carbohydrates (10–30% of total caloric intake) and low in fat (18–40% of total caloric intake) have demonstrated short-term effectiveness. These dietary approaches are thought to enhance satiety, leading to a reduction in overall caloric consumption [126]. However, adhering to these dietary practices can be particularly challenging in childhood and adolescence. The Mediterranean diet proves to be an effective compromise between acceptability and adequate nutritional intake [127], It is recommended as a protective dietary approach in the most recent guidelines issued by the Italian Society of Pediatric Endocrinology and Diabetology [128]. It has been demonstrated that adherence to the Mediterranean diet is associated with higher levels of circulating vitamin D, a nutrient particularly deficient in obese children and adolescents, due to the significant impact of fat accumulation on its metabolism [129,130]. An inverse association between circulating levels of 25-OH vitamin D and body composition has been observed [131]. Vitamin D deficiency is largely attributed to its lipophilic nature which causes the sequestration in adipose tissue, rendering it unavailable for biological functions [132]. Additionally, obesity is linked to reduced expression of genes that regulate the enzymes 25-hydroxylase and 1α-hydroxylase, both of which are critical for vitamin D metabolism. This leads to compromised production of 25-OH vitamin D in the liver and adipose tissue, resulting in lower serum vitamin D levels [133]. In addition, limited sun exposure, common in sedentary lifestyles associated with obesity, further reduces natural vitamin D synthesis [134]. Impaired renal function and increased vitamin D catabolism due to liver steatosis also contribute to lower 25-OH vitamin D levels, as demonstrated in a meta-analysis examining children with non-alcoholic fatty liver disease [135]. The effects of vitamin D deficiency go beyond its impact on bone health. Poor vitamin D status in obese children aggravates their metabolic profiles, increasing susceptibility to impaired glucose metabolism [130]. Vitamin D plays a pivotal role in insulin secretion and sensitivity, as well as systemic inflammatory response. It directly binds to vitamin D receptors in pancreatic β-cells to enhance insulin release and indirectly raises plasma calcium levels, promoting calcium-dependent insulin secretion [136,137]. The Italian Pediatric Society advises seasonal vitamin D supplementation for children and adolescents with limited sun exposure during the summer months, specifically from late autumn to early spring (November–April) or with specific risk factors, including obesity [138]. However, the effects of vitamin D supplementation on bone health in obese children and adolescents have yielded mixed findings. A recent meta-analysis evaluating the effectiveness of vitamin D supplementation in obese children and adolescents revealed that high doses of vitamin D benefit cardiovascular metabolism and improve insulin resistance; however, no significant impact on bone health was observed [133]. The absence of direct effects on bone health observed in the meta-analysis may be attributed to the unique characteristics of bone formation and growth during adolescence, a period of enhanced skeletal activity. Unlike in adults, parathyroid hormone (PTH) activity in adolescents is influenced by complex physiological mechanisms that are not necessarily tied to bone metabolism. Elevated PTH levels in this age group may reflect these intricate processes, which appear relatively independent of vitamin D supplementation [139]. On the other hand, a recent study conducted by Wang et al. investigated the effects of vitamin D3 supplementation on bone mass in obese pediatric patients. They found that vitamin D3 supplementation improved lipid profiles, glucose metabolism, as well as bone mass development in obese children [140].

Physical activity is essential for bone health [141]. Muscle and bone interact anatomically, mechanically, and via paracrine and endocrine signals [142], mediated by muscle-secreted factors like myokines [143]. Among these factors, irisin is crucial for bone metabolism, as it facilitates the differentiation of bone marrow stromal cells into mature osteoblasts [144]. Achieving an osteogenic response requires deformation of bones beyond their usual thresholds. However, there is limited evidence to determine which sport is most effective for obese children in simultaneously achieving weight loss and promoting bone modeling [141]. However, for children with obesity, joint pain, reduced mobility, and low self-esteem can be barriers to such activities [145].

**Figure 3:**
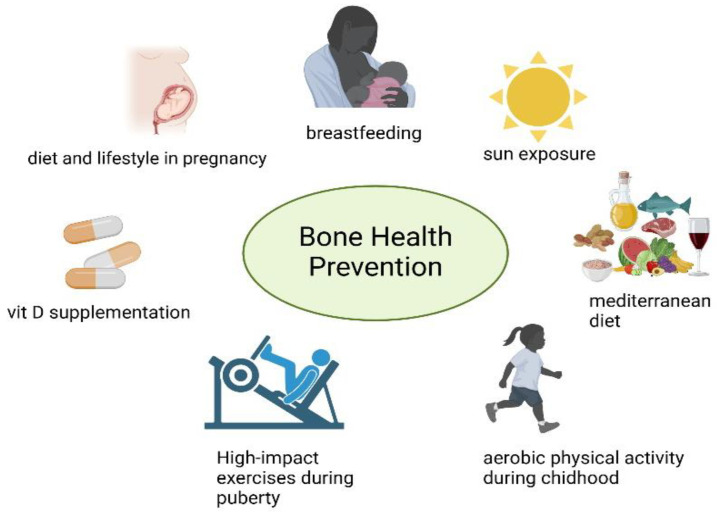
Promoting bone health involves a comprehensive approach starting from the preconception period and pregnancy, where maternal diet and lifestyle influence future health outcomes for both mother and child. Breastfeeding plays a key protective role, supported by evidence linking human milk’s composition to better weight regulation and bone health in early childhood. Vitamin D supplementation is crucial, particularly for children and adolescents with obesity, who are at higher risk of vitamin D deficiency due to reduced sun exposure, dietary insufficiency, and metabolic alterations. The Mediterranean diet is highlighted as an effective and sustainable dietary pattern, promoting higher vitamin D levels and providing essential nutrients such as calcium and phosphorus, necessary for bone mineralization. Physical activity, particularly high-impact exercises during puberty, enhances bone strength through mechanical stress and improves overall fitness. Aerobic activities during childhood, combined with resistance training in adolescence, play a significant role in fostering healthy bone development while addressing risk factors such as obesity and sedentary behavior.

Starting with low-impact exercises, like swimming or resistance training, helps promote bone health while reducing strain. Gradual progression to more intensive activities can improve fitness and resilience. Incorporating daily movement, such as walking to school or active family outings, and structured, enjoyable programs tailored to individual abilities can encourage consistent participation [146]. The findings underscore the importance of prevention that should begin as early as the prenatal stage, where maternal nutrition and vitamin D levels play a pivotal role in shaping the skeletal development of offspring. Extending into early childhood, breastfeeding and the adoption of balanced dietary practices, such as the Mediterranean diet, are essential to prevent excessive weight gain and for supporting bone mineralization. In children and adolescents already affected by obesity, targeted nutritional strategies are indispensable and supplementation of vitamin D should be considered. Diet must be accompanied by physical activity, the cornerstone of any intervention aimed at improving bone health in obese children and adolescents. Exercise not only enhances bone strength through mechanical loading but also counters sedentary behaviors that exacerbate skeletal fragility (Figure 3).

## 6. Impact of Severe Obesity Drug Treatments and Bariatric Surgery on Bone Health

Glucagon-like peptide 1 receptor agonists (GLP-1 RAs) and bariatric surgery represent new possibilities for the treatment of severe obesity [147].

GLP-1 RAs, including liraglutide, exenatide, dulaglutide, and semaglutide, reduce hunger by slowing gastric emptying and targeting the central nervous system [148,149,150,151,152].

Clinical trials on the effects of anti-obesity drugs on bone health are reported in Table 3.

Liraglutide is currently the sole GLP-1 receptor agonist approved in Europe for managing pediatric obesity in children and adolescents aged 12 years and above, with a BMI of 30 kg/m^2^ or greater, or 27 kg/m^2^ or greater if accompanied by comorbidities such as T2D or metabolic syndrome [153]. Liraglutide is given via subcutaneous injection, beginning at a dose of 0.6 mg/day and gradually increasing to a maximum of 3.0 mg/day. Several studies have highlighted the effects of GLP-1 on bone metabolism [154,155,156,157,158]. The administration of GLP-1 or its analog, exendin-4, was found to increase BMD and promote the expression of osteoblast markers. These effects were mediated by mechanisms such as activation of the Wnt pathway and an elevated OPG/RANKL ratio in normal, diabetic, and hyperlipidemic rats [154,155]. Exendin-4 also increases BMD by suppressing SOST/sclerostin expression in MLO-Y4 cells, resulting in elevated serum osteocalcin levels, reduced serum sclerostin levels, and increased femoral BMD in T2D rats [156]. In a study by Iepsen et al., involving 37 obese women, it was observed that treatment with long-acting liraglutide over 52 weeks resulted in a 16% increase in bone formation and helped prevent bone loss following weight reduction through a low-calorie diet [158]. Recently, a randomized clinical trial analyzed the effect of a 1-year intervention with either liraglutide (3.0 mg/day), exercise, or the combination of both on BMD in obese subjects undergoing weight loss compared with placebo [158].

The combination of liraglutide and exercise resulted in the most significant reductions in weight and body fat, while preserving hip, spine, and forearm BMD compared to the placebo group. In contrast, liraglutide alone caused a decrease in hip and spine BMD compared to both the placebo and exercise-only groups. These findings underscore the crucial role of physical activity in counteracting the negative impact of liraglutide on bone health during weight loss interventions [158].

The use of bariatric surgery (BS) has shown promising results in severe obese adolescents, with evidence of long-term efficacy and good safety [159]. Laparoscopic sleeve gastrectomy (SG) and Roux-en-Y gastric bypass (RYGB) are the most frequently performed procedures in adolescents. The maintenance of weight achieved after surgery is satisfactory from 3 years up to 12 years postsurgery [160,161]. Moreover, bariatric surgery’s metabolic impact is widely acknowledged, with rates of T2D remission reaching up to 86% in adolescents compared to 53% in adults [162]. The impact of BS on the skeleton during a crucial phase of bone growth and development continues to be a major concern. BS has been associated with reductions in BMD across various skeletal sites. Mitra et al., in a systematic review and meta-analysis, revealed that RYGB and sleeve gastrectomy SG were associated with substantial reductions in lumbar BMD Z-scores [163]. These procedures also led to elevated markers of bone resorption, such as C-terminal telopeptide, indicating intensified bone turnover. These data highlight the importance of pharmacological treatments like GLP-1 receptor agonists as a promising avenue for weight management but require careful consideration of their effects on skeletal health. Combining these treatments with structured physical activity may prevent the reductions in bone density observed during weight loss. Similarly, while bariatric surgery offers substantial weight reduction benefits, its long-term skeletal risks necessitate a rigorous postoperative regimen of supplementation, dietary management, and physical activity.

**Table 3:** Clinical trials on the effects of anti-obesity drugs on bone health.

Phases	Clinical Trials.Gov	Start Date of Trial	Drug	Subjects	Measurements	Effects on Bone Health	Adverse Effects	References
III	NCT02918279	September 2016	Liraglutide	Obese adolescents and patients with T2DM	BMI	Not investigated	Nausea, vomiting, diarrhea	[149]
III	NCT00097500	July 2019	Exenatide	Metformin-treated patients with T2DM	BMD, ALP, Ca	BMD, serum markers of bone metabolism, and calcium homeostasis were unaffected by exenatide treatment	Nausea	[150]
N/A	NCT01147627	August 2010	Exenatide	62 patients with T2D, randomized into 3 groups: exenatide, insulin, pioglitazone	BMD, CTX, OC, TRAcP5b	No impact on bone turnover markers or BMD	Nausea	[151]
III	NCT01648582	July 2012	Exenatide Dulaglutide	Patients with T2DM randomized into four groups: exenatide group, dulaglutide group, insulin glargine group, placebo	BMD	Exenatide group: increased total hip BMD. Dulaglutide group: decreased femoral neck BMD	Not investigated	[152]
N/A	NCT02094183	September 2019	Liraglutide	Obese women	BMD, CTX-1, P1NP	BMD increase	Not investigated	[157]
N/A	NCT04122716 secondary analysis	August 2016	Liraglutide	Obese adults randomized into 4 groups: exercise alone, liraglutide alone, the combination, or placebo	BMD at the hip, lumbar spine, and distal forearm	BMD decreased in liraglutide group	Not investigated	[158]

**Abbreviations:** BMI, Body Mass Index; BMD, Bone Mineral Density; CTX-1, C-terminal telopeptide of type 1 collagen; P1NP, N-terminal propeptide of type 1 procollagen; T2DM, Type 2 Diabetes Mellitus; ALP, Alkaline Phosphatase; Ca, calcium; OC: Osteocalcin; TRAcP5b, Tartrate-resistant acid phosphatase 5b; N/A, Not Available.

## 7. Conclusions

The intricate relationship between childhood obesity and bone health reflects a balance between mechanical loading benefits and the detrimental effects of chronic inflammation, suboptimal nutrition, and altered endocrine signals. Although increased body weight can initially yield higher bone mass, this apparent advantage is undermined by poor trabecular architecture, micronutrient deficiencies, and inflammatory pathways that favor bone resorption. Syndromic forms of obesity and excess weight due to eating disorders, including neurodevelopmental conditions such as autism, add further complexity, often compounding bone fragility through hormonal dysregulations, insufficient vitamin intake, and reduced physical activity. Innovations in treatment of severe obesity, ranging from GLP-1 RAs to BS, underscore both the promise and the inherent risks to skeletal integrity. Pharmacological agents may offer balanced reductions in adiposity and preservation of bone mass if integrated with exercise regimens. BS can achieve dramatic weight loss and metabolic improvements but frequently leads to bone demineralization and heightened fracture risk, underscoring the need for structured follow-up and targeted supplementation.

Prevention through the promotion of healthy lifestyles, such as a diet based on the principles of the Mediterranean diet and the practice of regular physical activity, remains the main objective to be pursued in order to preserve bone health. Future research must prioritize the development of longitudinal studies to better understand the short- and long-term effects of pharmacological therapies and BS on skeletal health. These studies should focus on identifying critical windows for intervention and defining strategies to mitigate bone fragility. Innovations in personalized medicine hold promise for integrating individualized nutrition plans, tailored exercise programs, and optimized vitamin supplementation. Additionally, exploring molecular pathways involved in obesity-induced bone alterations, such as the role of adipokines and inflammatory mediators, could uncover novel therapeutic targets. Advanced imaging technologies and biomarkers should be leveraged to monitor changes in bone quality more accurately over time. A multidisciplinary effort is essential, bringing together pediatricians, endocrinologists, nutritionists, and exercise specialists to create holistic, sustainable strategies that address the dual challenges of obesity and bone health. These advancements will ultimately contribute to reducing the burden of obesity-related skeletal complications and improving long-term outcomes for children and adolescents.

## Figures and Tables

**Table 1:** In vitro and in vivo studies on the impact of adipose tissue and obesity on bone.

Author(s)	Year	Study Type	Model/System	Key Findings	References
Da Silva et al.	2016	In vitro	Bone marrow MSCs	Inflammatory bone marrow microenvironment drives MSCs toward adipogenesis	[12]
Cortez et al.	2013	In vitro/in vivo	Bone marrow MSCs isolated from the femurs of Wistar rats subjected to HFD	NF-κB activation increased, while PPAR-γ expression decreased, signaling a shift toward an inflammatory state and reduced adipogenesis; high levels of pro-inflammatory cytokines	[13]
Patsch et at.	2011	In vivo	Mouse model of HFD-induced obesity	Alterations in trabecular microarchitecture, even after short periods of exposure	[14]
Lam et al.	2000	In vitro	Murine macrophages	TNF-α combined with minimal levels of RANKL enhances osteoclast differentiation through the activation of NF kappa-B and JNK pathways	[15]
Azuma et al.	2000	In vitro	Osteoclast progenitors in cell cultures	TNF-α directly induced the formation of multinucleated TRAP-positive osteoclasts	[16]
Kim et al.	2005	In vitro	Hematopoietic precursors derived from TRANCE-null, RANK-null, or TRAF6-null mice	Differentiation into osteoclasts when stimulated with TNF-α and TGF-β	[17]
Glass et al.	2011	In vitro	MDSCs harvested from mice three days after exposure to an adjacent fracture	TNF-α promotes MDSC migration and osteogenic differentiation at low concentrations	[18]
Li et al.	2021	In vivo/in vitro	BMSCs isolated from both WT and IL-6 KO mice	IL-6 drives MSC senescence and trabecular bone loss through STAT3/p53/p21 signaling	[19]
Kim et al.	2006	In vitro	Human peripheral blood mononuclear cells cultured in the presence M-CSF and MCP-1	MCP-1 induces TRAP(+)/CTR(+) multinuclear cells that represent an arrested stage in osteoclast differentiation	[20]
Cornish et al.	2002	In vitro/in vivo	Fetal rat osteoblasts, mouse bone marrow cultures, isolated chondrocytes	Leptin directly promotes bone cell function by stimulating osteoblast and chondrocyte proliferation and inhibiting osteoclastogenesis	[21]
Xin et al.	2011	In vitro	C2C12 myotubes, a murine muscle cell line	APPL1 specifically regulated adiponectin-induced p38 MAPK activation but had no effect on p38 MAPK phosphorylation in response to TNF-α	[23]
Luo et al.	2006	In vitro	Human osteoblasts	Recombinant adiponectin modulated RANKL and OPG expression in human osteoblasts in a dose- and time-dependent manner	[24]
Tu et al.	2011	In vitro/in vivo	RAW264.7 cells. Femurs isolated from double-labeled transgenic mice (mBSP9.0Luc/β-ACT-EGFP) transplanted into adiponectin KO mice and WT mice	Adiponectin suppressed RANKL-induced osteoclast formation from RAW264.7 cells by downregulating key osteoclastogenic regulators	[25]
Bartell et al.	2011	In vivo	Ob/Ob mice	Intracerebroventricular leptin increased pro-osteogenic gene expression in bone marrow and decreased expression of genes associated with osteoclastogenesis	[26]
Williams et al.	2011	In vivo	Db/Db mice	Db/Db mice showed reduced bone mass, strength, and formation rates compared to WT mice	[27]
Steppan et al.	2000	In vivo	Ob/Ob mice	Leptin administration in Ob/Ob mice increased bone length, density, and mass	[28]
Naot et al.	2016	In vivo	WT and adiponectin-KO mice	Adiponectin-KO mice showed reduced body fat, decreased BMD, and lower cortical and trabecular bone volume compared to WT mice	[29]
Wang et al.	2013	In vivo	Femur and vertebra in sham-operated and ovariectomized adiponectin-KO mice	Adiponectin-KO mice showed no changes in BMD but exhibited increased ALP activity, osteoclast numbers, and enhanced osteogenic differentiation of MSCs, with higher Runx2 and Osterix expression	[31]
Lecka-Czernik et al.	2015	In vivo	C57BL/6 mice males fed with HFD or regular diet	HFD-induced obesity in C57BL/6 male mice increased bone mass compared to controls	[32]
Ootsuka et al.	2015	In vivo	Hyperphagic and obese rat model	Hyperphagic-induced obesity in rats with normal glycemic control increased osteoclastogenesis. These effects were linked to elevated TNF-α levels and NF-κB activation	[33]
Feng et al.	2016	In vivo	IL-6(−/−) mice and WT mice	HFD-induced obesity reversed IL-6-deficiency-related bone remodeling abnormalities in IL-6(−/−) mice	[34]
Fujita et al.	2012	In vivo	Male C57BL/6J with normal and HFD	In early stages, diet-induced obesity reduced trabecular bone density due to increased adipocytes and trabecular deterioration	[35]
Silva et al.	2019	In vivo	Mice from the Large-by-Small advanced intercross line (F34 generation)	Bone size and strength correlated with body mass, but this relationship was weaker in HFD-fed mice compared to low-fat diet-fed mice	[36]
Yamasaki et al.	2016	In vivo	HFD-fed mice	In osteoclasts, AACS mRNA expression was significantly upregulated by IL-6, linking ketone body metabolism, AACS, and osteoclast activity	[37]
Chaplin et al.	2015	In vivo	C57BL/6J mice	Conjugated linoleic acids reduced tibia weight with minimal impact on bone markers, while calcium, alone or with conjugated linoleic acids, preserved bone weight, enhanced bone formation gene expression	[38]
Ip et al.	2009	In vivo	Obese (fa/fa) and lean (Fa/Fa) male Zucker rats	Obese Zucker rats (*fa*/*fa*) had normal bone ash levels despite reduced bone size	[39]

**Abbreviations:** AACS: Acetoacetyl-CoA Synthetase, ACT-EGFP: Beta-Actin Enhanced Green Fluorescent Protein, APN: Adiponectin, APPL1: Adaptor Protein Containing PH Domain PTB Domain and Leucine Zipper Motif 1, BMD: Bone Mineral Density, CTR: Calcitonin Receptor, HFD: High-Fat Diet, IL-6: Interleukin-6, JNK: c-Jun N-terminal Kinase, KO: Knockout, M-CSF: Macrophage Colony-Stimulating Factor, MAPK: Mitogen-Activated Protein Kinase, MCP-1: Monocyte Chemoattractant Protein-1, MDSC: Muscle-Derived Stromal Cell, MSC: Mesenchymal Stem Cell, NF-κB: Nuclear Factor Kappa-B, Ob/Ob: Obese Mouse Model, OPG: Osteoprotegerin, PPAR-γ: Peroxisome Proliferator-Activated Receptor Gamma, RANK: Receptor Activator of Nuclear Factor Kappa-B, RANKL: Receptor Activator of Nuclear Factor Kappa-B Ligand, Runx2: Runt-related Transcription Factor 2, STAT3: Signal Transducer and Activator of Transcription 3, TGF-β: Transforming Growth Factor Beta, TNF-α: Tumor Necrosis Factor Alpha, TRAF6: TNF Receptor-Associated Factor 6, TRANCE: TNF-Related Activation-Induced Cytokine, TRAP: Tartrate-Resistant Acid Phosphatase, WT: Wild-Type.

**Table 2:** Human studies on impact of obesity on bone health.

Author(s)	Year	Countries	Sample Size and Characteristics	Methodology	Type of Study	Key Findings	References
Leonard et al.	2004	USA	32 non-obese and 103 obese subjects (4–20 years)	Whole-body and BMC were measured using DXA	Cross-sectional study	Obesity was linked to advanced maturation and higher lean mass for height	[42]
Kessler et al.	2013	USA	913,178 patients (2 to 19 years)	BMI was used to classify patients into 5 weight categories, and records were analyzed for lower extremity fractures	Cross-sectional study	Higher BMI was associated with an increased risk of foot, ankle, knee, and leg fractures, especially in children aged 6–11 years	[47]
Gil-Cosano et al.	2019	Spain	55 children (10.2 ± 1.2 years)	Assessments of body composition by DXA, inflammatory markers (IL-6, IL-1β, TNF-α), and muscular fitness	Cross-sectional study	IL-6, VEGF, TNF-α, and IL-1β show a strong correlation with bone mass	[48]
Erazmus et al.	2022	Poland	70 children and adolescents with overweight and obesity (7.0 to 17.8 years) and 35 age-matched controls	OGTT, atherogenic and insulin resistance indices	Case–control study	Overweight and obese children had lower sRANKL levels and a higher OPG/sRANKL ratio	[49]
Brunetti et al.	2020	Italy	111 obese subjects (12.21 ± 3.71 years) and 45 controls	AD-SoS-Z and BTT-Z scores by QUS, LIGHT serum levels, osteoclastogenesis by culturing PBMCs with or without the addition of anti-LIGHT antibody	Case–control study	BMI-SDS negatively correlated with AD-SoS-Z and BTT-Z scores. Elevated serum LIGHT levels and increased LIGHT expression on monocytes, CD3+ T-cells, and neutrophils were observed in obese subjects	[50]
Dimitri et al.	2015	UK	18 lean children and 18 obese participants	HR-pQCT	Case–control study	Obese children showed lower radial cortical porosity and pore diameter, reduced tibial trabecular thickness, and higher trabecular number	[51]
Ugur-Altun et al.	2005	Turkey	50 obese subjects (31 ± 8 years) and 24 lean controls (30 ± 7 years)	HOMA-IR, OPG	Cross-sectional study	Obese individuals with higher insulin resistance had lower OPG levels compared to those with lower insulin resistance and lean controls	[56]
Suliburska et al.	2013	Poland	Obese subjects (12–18 years)	Anthropometrical measurements and blood biochemical analyses	Cross-sectional study	Higher OPG levels and HOMA-IR indices in obese adolescents were positively correlated, linking elevated OPG to insulin resistance	[57]
Oh et al.	2020	Korea	232 overweight and obese children (6–15 years)	Anthropometric and laboratory data and the degree of MASLD	Cross-sectional study	Advanced bone age is more common in obese children, particularly with higher BMI, insulin resistance, metabolic syndrome, and severe MASLD, along with lower HDL cholesterol levels	[59]
Clark et al.	2006	UK	3503 children assessed at age of 9.9 years and followed up at 11.8 years	DXA	Cross-sectional and prospective cohort study	Fat mass stimulates bone growth in boys and prepubertal girls but diminishes or reverses in later pubertal stages, likely due to puberty’s impact	[61]
Fornari et al.	2013	USA	992 children (1–13 years)	Evaluation of BMI and BMI-for-age percentiles. Fracture classification	Retrospective cohort study	Obese children have a greater risk of sustaining a lateral condyle fracture and, when these fractures occur, they are often more severe injuries	[62]

**Abbreviations:** BMC: Bone Mineral Content, DXA: Dual-Energy X-ray Absorptiometry, DXA: Dual-Energy X-ray Absorptiometry, HDL: High-Density Lipoprotein, HOMA-IR: Homeostasis Model Assessment of Insulin Resistance, HR-pQCT: High-Resolution Peripheral Quantitative Computed Tomography, IL-1β: Interleukin-1 beta, IL-6: Interleukin-6, MASLD: Metabolic Associated Steatotic Liver Disease, OGTT: Oral Glucose Tolerance Test, OPG: Osteoprotegerin, PBMCs: Peripheral Blood Mononuclear Cells, sRANKL: Soluble Receptor Activator of Nuclear Factor Kappa-B Ligand, TNF-α: Tumor Necrosis Factor Alpha, VEGF: Vascular Endothelial Growth Factor.

## Abbreviations

The following abbreviations are used in this manuscript:

ALP	Alkaline Phosphatase
AN	Anorexia Nervosa
APPL1	Adaptor Protein, Phosphotyrosine Interacting with PH Domain and Leucine Zipper1
ARFID	Avoidant/Restrictive Food Intake Disorder
ASD	Autism Spectrum Disorder
BMC	Bone Mineral Content
BMD	Bone Mineral Density
BMSC	Bone Mesenchymal Stem Cell
BMI	Body Mass Index
BN	Bulimia Nervosa
Ca	Calcium
CTX	C-terminal Telopeptide
DXA	Dual-Energy X-ray Absorptiometry
DPD	Deoxypyridinoline
GH	Growth Hormone
GLP-1	Glucagon-like Peptide-1
GLP-1R	Glucagon-like Peptide-1 Receptor
HFD	High-Fat Diet
HOMA-IR	Homeostatic Model Assessment of Insulin Resistance
IGF-1	Insulin-like Growth Factor 1
IL-6	Interleukin-6
LIGHT	Lymphotoxin-like Inducible Protein that Competes with Glycoprotein D for Herpesvirus Entry Mediator on T Cells
MAPK	Mitogen-Activated Protein Kinase
MBS	Metabolic and Bariatric Surgery
MCP-1	Monocyte Chemoattractant Protein-1
N/A	Not Available
OC	Osteocalcin
OPG	Osteoprotegerin
PA	Physical Activity
P1NP	Procollagen Type 1 N-terminal Propeptide
Pica	Persistent Ingestion of Non-food Substances
PPARγ	Peroxisome Proliferator-Activated Receptor Gamma
PTH	Parathyroid Hormone
QUS	Quantitative Ultrasound
RANK	Receptor Activator of Nuclear Factor Kappa-B
RANKL	Receptor Activator of Nuclear Factor Kappa-B Ligand
RYGB	Roux-en-Y Gastric Bypass
SG	Sleeve Gastrectomy
STAT3	Signal Transducer and Activator of Transcription 3
T2DM	Type 2 Diabetes Mellitus
TGFβ	Transforming Growth Factor-beta
TNF-α	Tumor Necrosis Factor-alpha
TRAcP5b	Tartrate-resistant Acid Phosphatase 5b
WHO	World Health Organization
